# End expiratory oxygen concentrations to predict central venous oxygen saturation: an observational pilot study

**DOI:** 10.1186/1471-227X-6-9

**Published:** 2006-09-20

**Authors:** Alan E Jones, Karl Kuehne, Michael Steuerwald, Jeffrey A Kline

**Affiliations:** 1 Department of Emergency Medicine, Carolinas Medical Center, Charlotte, NC, USA

## Abstract

**Background:**

A non-invasive surrogate measurement for central venous oxygen saturation (ScVO2) would be useful in the ED for assessing therapeutic interventions in critically ill patients. We hypothesized that either linear or nonlinear mathematical manipulation of the partial pressure of oxygen in breath at end expiration (EtO2) would accurately predict ScVO2.

**Methods:**

Prospective observational study of a convenience sample of hemodialysis patients age > 17 years with existing upper extremity central venous catheters were enrolled. Using a portable respiratory device, we collected both tidal breathing and end expiratory oxygen and carbon dioxide concentrations, volume and flow on each patient. Simultaneous ScVO2 measurements were obtained via blood samples collected from the hemodialysis catheter. Two models were used to predict ScVO2: 1) Best-fit multivariate linear regression equation incorporating all respiratory variables; 2) MathCAD to model the decay curve of EtO2 versus expiratory volume using the least squares method to estimate the pO2 that would occur at <20% of total lung capacity.

**Results:**

From 21 patients, the correlation between EtO2 and measured ScVO2 yielded R^2 ^= 0.11. The best fit multivariate equation included EtCO2 and EtO2 and when solved for ScVO2, the equation yielded a mean absolute difference from the measured ScVO2 of 8 ± 6% (range -18 to +17%). The predicted ScVO2 value was within 10% of the actual value for 57% of the patients. Modeling of the EtO2 curve did not accurately predict ScVO2 at any lung volume.

**Conclusion:**

We found no significant correlation between EtO2 and ScVO2. A linear equation incorporating EtCO2 and EtO2 had at best modest predictive accuracy for ScVO2.

## Background

The mixed venous oxygen saturation (SVO2) is widely used in clinical practice to assess shock states and the physiologic response to resuscitation [1,2]. The central venous oxygen saturation (ScVO2) measurement correlates closely with SVO2 and when necessary can be substituted as a less invasive surrogate to the SVO2 [3]. When combined with other parameters, the use of ScVO2 measurements for guiding resuscitation has been shown in one study to improve mortality in patients with septic shock [4]. Both the SVO2 and ScVO2 measurements require central venous cannulation and a catheter to be placed in either the right atrium or pulmonary artery, thus limiting the feasibility of this measurement in the emergency department (ED) [5]. Accordingly, a non-invasive method to measure SVO2 and ScVO2 would be useful for critically ill ED patients requiring resuscitation.

Because the percentage of erythrocyte hemoglobin with bound oxygen varies with the partial pressure of oxygen in plasma according to a well-defined allosteric curve, the partial pressure of oxygen dissolved in plasma generally can be used with reasonable accuracy to predict the percentage of hemoglobin saturated with oxygen. Normally there is rapid equilibration of the partial pressure of oxygen between the alveolus and corresponding pulmonary arteriole at all intervals of the respiratory cycle. It would then seem logical that the partial pressure of oxygen in central venous blood would correlate directly with the nadir partial pressure of oxygen in deep expired breaths and thus provide a non-invasive method of estimating the ScVO2. The hypothesis of the present study states that the partial pressure of expired oxygen in end tidal breaths (EtO2) will correlate with ScVO2.

## Methods

We performed an observational proof of concept study of a convenience sample of ambulatory hemodialysis patients. This study was approved by the Institutional Review Board and Privacy Board of the Carolinas HealthCare System and all patients gave written informed consent to participate. The recommendations of the most current Helsinki Declaration were followed.

Patients were recruited at the time they presented for routine hemodialysis at the kidney dialysis unit of Carolinas Medical Center, a large urban tertiary referral center with > 800 inpatient beds and an ED census of > 110,000 visits per year. The inclusion criteria for the study were age > 17 years and central venous hemodialysis catheter in either the internal jugular or subclavian vein. Exclusion criteria were a known heart condition resulting in either right to left or left to right cardiac shunting or non-invasive peripheral arterial oxygen saturation < 90%.

After subject identification and informed consent, we collected breaths using standardized protocol [6]. Briefly, just prior to initiation of hemodialysis and at the time the nurse accessed the central venous catheter, 2 mL of venous blood was obtained in a sodium heparin syringe, immediately placed on ice for analysis. Then, while in semi-Fowler's position, and wearing nose clips, patients breathed into a duckbill-shaped mouthpiece in airtight connection with the airflow transducer. A research assistant provided help to the patient as needed. Patients delivered a sharp, rapid, deep exhalation to a maximum endpoint, starting from a midpoint of tidal breathing (i.e., not delivered after a sigh inspiration) followed by a few normal breaths, and then a 30 second period of tidal breathing. This sequence was repeated three more times, yielding four deep exhalations and three 30-second samples of tidal breathing. At the time of enrollment patients were breathing room air.

### Measurements

#### Breath collection

The device used to measure expired volume, expired partial pressure of carbon dioxide (EtCO2) and EtO2 was constructed using commercially available components. Expired volume was quantitated by a pneumotach, airflow transducer (TSD127, Biopac Systems Inc., Santa Barbara, CA,) connected to a distal polycarbonate tube of same diameter fitted with a 4 millimeter lure-lock port for aspiration of gases. A low-resistance, 0.2 uM antimicrobial filter (1644 Intersurgical Inc., Liverpool, NY) was placed between the pneumotach and a duckbill-style mouthpiece (1565, Hudson Respiratory Care Inc., Temecula, CA). Expired carbon dioxide and oxygen were measured in side-stream fashion via separate vacuum pumps that each aspirated 12 milliliters/minute through 1 meter long, 3 millimeter internal diameter polyethylene tubing. Carbon dioxide and oxygen partial pressures were quantified in real time by infrared absorptiometry and paramagnetic deviation (Biopac Systems Inc., Santa Barbara, CA). Both sensors were calibrated against two dry reference gases before each patient, and readings of reference gases were repeated immediately after data were collected from each patient to evaluate for calibration stability. The airflow transducer was tested against a volumetric calibration syringe (AFT 26 2L, Biopac Systems Inc., Santa Barbara, CA) immediately before and after each patient. Airflow, expired volume, continuous tracings of expired CO2 and O2 were recorded at body temperature, saturated with water and at ambient pressure, and were archived digitally using commercial analog-to-digital converter and commercial software (MP-100, and AcqKnowledge ACK100W, respectively, Biopac Instruments Inc., Santa Barbara, CA). For each measurement (flow, volume, EtO2 and EtCO2) the average of the four different deep exhalation was used for data analysis.

#### Blood gas analysis

Iced blood samples drawn from the hemodialysis catheters were transported within 3 minutes to a Stat Profile Ultra Analyzer (Nova Biomedical, Waltham, MA). Samples underwent analysis for measured percent oxygen saturation via co-oximetry. Samples were analyzed in duplicate and the average of the two readings was used for data analysis.

### Data analysis

**Figure 1:**
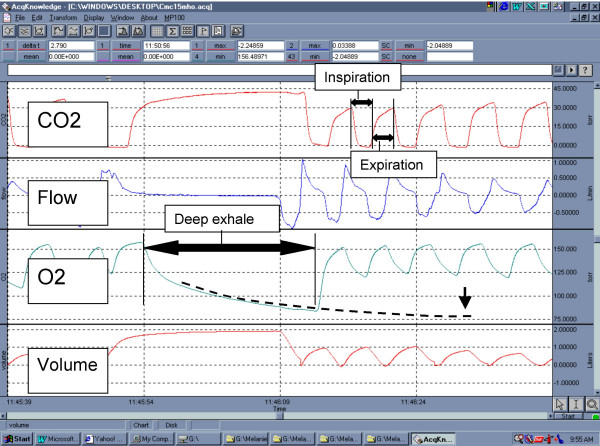
An example of the raw data that was analyzed from exhaled breath measurements. The third panel represents the partial pressure of expired oxygen. The long exhalation represents a voluntary deep exhalation, done on command as part of the data collection protocol. The superimposed dotted line hypothetically represents the regression equation where the zero slope portion, denoted by the arrow, represents the steady-state estimate of the partial pressure of oxygen in mixed venous blood. Inspiratory, expiratory and deep exhale cycles are annotated on the figure. CO2 – carbon dioxide; Vol – volume; O2 – oxygen.

The breath measurements obtained were compared in a multivariate linear regression model to determine an equation that would best predict the ScVO2 (Microsoft Excel, Redmond, WA). The potential coefficients of the equation included EtO2, EtCO2, flow or volume. Figure 1 shows an example of the raw data that was analyzed. The third panel represents the partial pressure of expired oxygen. The long exhalation represents a voluntary deep exhalation, done on command as part of the data collection protocol. The superimposed dotted line hypothetically represents the regression equation where the zero slope portion, denoted by the arrow, represents the steady-state estimate of the partial pressure of oxygen in mixed venous blood. Additional analysis included modeling the decay curve of EtO2 versus expiratory volume using the least squares method to estimate the EtO2 that would occur at 5% increments between 0 and 20% of total lung capacity (MathCAD, Mathsoft, Cambridge, MA). Total lung capacity was estimated using standard curves based upon height, gender and age [7]. A sample size of 20 patients was estimated in order to generate sufficient raw breath data for extrapolation and modeling.

## Results

**Figure 2:**
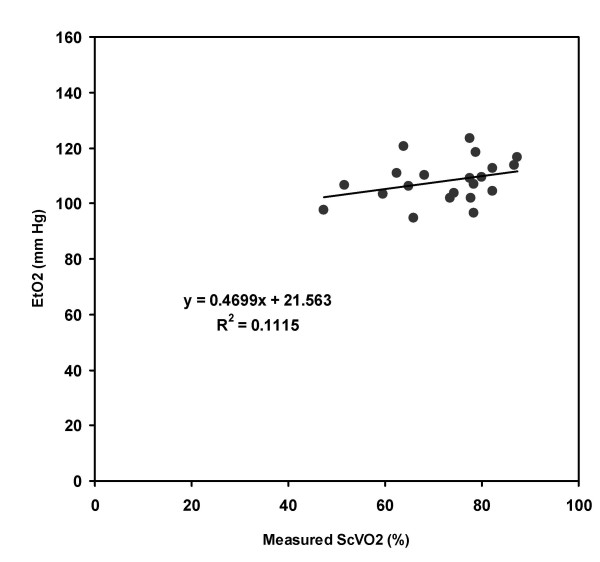
Regression analysis of the EtO2 versus measured ScVO2. The Y-axis values were the average of four deep exhalation end tidal partial pressure of oxygen measurements and the X-axis values were the average of two duplicate oxygen saturation measurements of central venous blood specimens obtained from a hemodialysis catheter. EtO2 – end-tidal oxygen; ScVO2 – central venous oxygen saturation.

**Figure 3:**
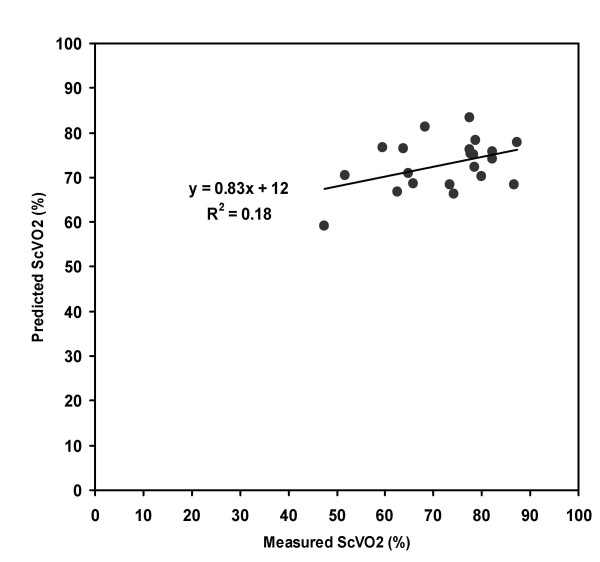
Regression analysis of the predicted ScVO2 versus measured ScVO2. The Y-axis values were derived from the equation Predicted ScVO2 = -78.1 + 1.2(EtCO2) + 0.95(EtO2), and the X-axis values were the average of two duplicate oxygen saturation measurements of central venous blood specimens obtained from a hemodialysis catheter. EtO2 – end-tidal oxygen; EtCO2 – end-tidal carbon dioxide; ScVO2 – central venous oxygen saturation.

Twenty-one patients were enrolled over a 6 month period in 2004–2005. The average age was 51.4 years, 60 % were male, and the average hemoglobin concentration was 10.9 grams/deciliter (g/dl). No patients had a hemoglobin of < 9.5 g/dl. Figure 2 shows that there was no significant correlation between EtO2 and measured ScVO2 with an R^2 ^= 0.11. The best fit multivariate regression equation was ScVO2 = -78.1 + 1.2(EtCO2) + 0.95(EtO2). When this equation was solved for ScVO2, as shown in Figure 3, there was no significant correlation between observed and predicted ScVO2 values with an R^2 ^= 0.18, (P = 0.057 with power to detect 5% difference), standard error on slope 0.4, standard error for predicted ScVO2 value = 2.3. When solved for ScVO2, the equation yielded a mean absolute difference from the measured ScVO2 of 8 ± 6% (range -18 to +17%). The predicted ScVO2 value was within ± 10% of the actual measured value for 12/21 (57%) of the patients. Table 1 shows the breath measurements for all patients. Least-squares modeling of the EtO2 decay curve did not predict ScVO2 at any lung volume with any reasonable degree of accuracy (e.g. >50% of estimates at all lung volumes tested were more than 50% off of the target ScVO2 value).

**Table 1:** Physiologic breath and blood measurements of all patients.

Gender	Average Peak EtCO2 (mmHg)	Average Minimum EtO2 (mmHg)	Average Tidal Volume (L)	Average Peak Flow (L/sec)	Deep Exhaled Maximum EtCO2 (mmHg)	Deep Exhaled Minimum EtO2 (mmHg)	Average Measured ScVO2 (%)	Predicted ScVO2 (%)
F	28	107	0.10	0.25	38	99	63	67
F	35	115	0.21	0.32	38	111	82	74
F	47	97	0.23	0.14	52	79	78	72
M	29	125	0.40	0.13	38	105	77	76
M	40	108	0.50	0.10	41	107	78	75
M	47	101	0.10	0.10	44	106	82	76
M	36	111	0.51	0.55	40	99	80	70
F	39	106	0.20	0.20	37	107	73	68
F	45	100	0.12	0.13	49	93	78	75
M	38	116	0.40	0.40	42	104	87	78
F	46	112	0.25	0.30	50	105	77	83
M	21	123	0.30	0.60	33	114	47	59
M	37	114	0.30	0.25	40	106	52	70
M	32	123	0.33	0.34	40	110	64	76
M	44	100	0.21	0.32	45	100	66	69
F	33	109	0.23	0.50	32	113	74	66
M	31	116	0.30	0.40	34	103	86	68
M	37	111	0.34	0.50	40	107	65	71
M	45	108	0.24	0.15	50	97	60	77
M	35	122	0.14	0.33	43	114	79	78
M	40	123	0.41	0.43	42	114	68	81

EtO2 – end-tidal oxygen; EtCO2 – end-tidal carbon dioxide; ScVO2 – central venous oxygen saturation; L – liters; mm Hg – millimeters of mercury; sec – second; M – male; F – female.

## Limitations

This report has several limitations to be addressed. First, because this was a feasibility study the sample size is small and which could contribute to an inaccurate estimation of the true predictive ability of exhaled breath measurements. Second, the patient population we studied were all hemodialysis patients. We chose hemodialysis patients because the ideal subjects for this study were ambulatory, not acutely ill, and had an indwelling central venous catheter that could be accessed for blood collection. We performed the measurements at the time of their routine dialysis so it is possible that sub-clinical pulmonary edema was present and lead to inaccurate exhaled oxygen measurements. To the best of our knowledge none of the patients that we enrolled had upper extremity arterial-venous fistulas which could lead to shunting and confounding measurements. Additionally, expired gases were measured in side-stream fashion which could have resulted in inaccurate measurements. The deep exhaled pO2 curve, measured by mainstream sampling with a rapid response oxygen probe andfittedbythe least squaresmethod may yield more accurate and precise estimations of ScVO2. Finally, we measured ScVO2 ex vivo and it is possible if we had used a continuous central venous oximetry catheter or pulmonary artery catheter the results may have been different.

## Discussion

Development of an accurate and non-invasive method of measuring systemic oxygen balance would be extremely useful in the evaluation and management of critically ill patients in the ED. In this study we investigated the use of end expiratory breath measurements to predict central venous oxygen saturation. The best predictive equation we derived performed only modestly. We did not find sufficient predictive accuracy to justify further investigation of this method.

The importance of developing non-invasive methods of identifying and quantifying shock as well as guiding the resuscitation of critically ill patients is evidenced by the recent number of publications touting potential new methods and devices for these purposes. Impedance cardiography [8], sublingual capnometry [9], near infrared spectrometry to measure tissue oxygen hemoglobin saturation [10,11], trancutaneous oxygen and carbon dioxide tensions [12], vital signs [13] and combinations of these measurements [14] have all been reported to have value in identification and monitoring of shock. To our knowledge, no non-invasive technologies have gained widespread use in clinical practice for monitoring shock.

In this study we evaluated a relatively simplistic idea, that expired breath concentrations of oxygen or carbon dioxide would predict central venous oxygen saturation. These breath measurements are easy to obtain, non-invasive, not stressful for patients, and could be performed in spontaneously breathing or mechanically ventilated patients. This type of measurement would be ideal for evaluating and monitoring patients in an emergency department where more complex and invasive monitoring is often not feasible [5]. Unfortunately, we were unable to show any consistent or convincing relationship between exhaled breath measurements and central venous oxygen saturation.

## Conclusion

We found no significant correlation between EtO2, measured by side-stream oximetry and ScVO2. A linear equation incorporating EtCO2 and EtO2 had at best modest predictive accuracy. Least-squares extrapolation of the expired EtO2 curve to low lung volumes produced erroneous estimations of ScVO2. We conclude that side-stream EtO2 measurements cannot be used in a straight-forward mathematical model to estimate ScVO2 at the bedside.

## Competing interests

Dr. Kline is co-founder and owns stock in Breathquant Incorporated. Dr. Kline is the inventor on a US patent for using breath measurements to guide resuscitation.

All the other authors declare that they have no competing interests.

## Authors' contributions

AEJ and JAK designed the study. AEJ, KK, MS, and JAK collected the data. AEJ and JAK analyzed the data. AEJ drafted the manuscript and all authors contributed to the final version.

## Pre-publication history

The pre-publication history for this paper can be accessed here:


